# Supplementary material for: Large-scale self-organization of reconfigurable topological defect networks in nematic liquid crystals
Source: Nat Commun. 2016 Nov 7;7:13238. doi: 10.1038/ncomms13238 (PMC5103064; doi:10.1038/ncomms13238)
Supplement: Supplementary Information — Supplementary Figures 1-6 [file ncomms13238-s1.pdf]

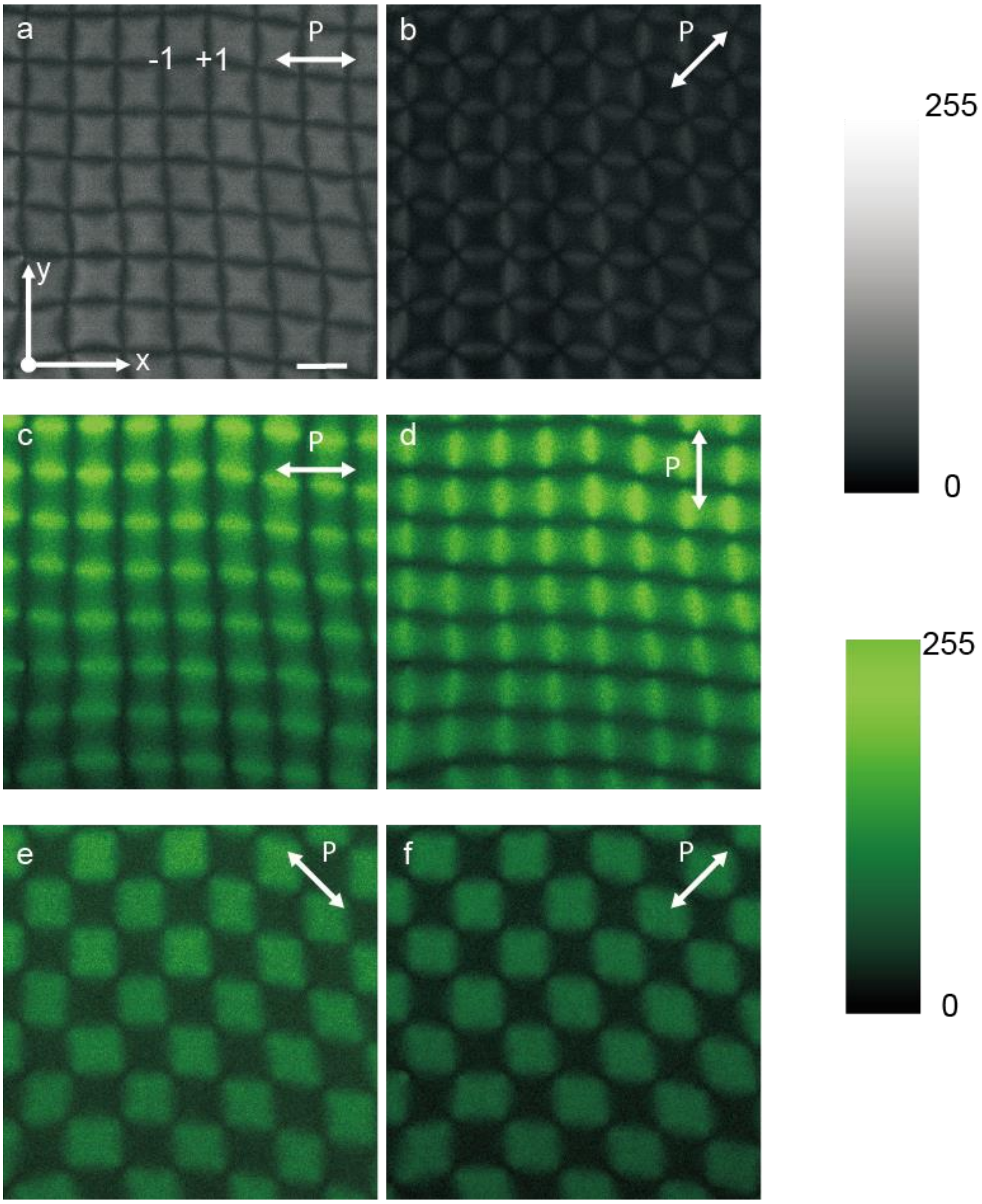

Supplementary Figure 1 : Images of grid-like texture obtained by fluorescent confocal polarizing microscopy (FCPM). Cross sectional images of the G state are taken at the middle height of the cell. (a) and (b) are obtained under UV transmission mode. (c)-(f) FCPM images taken with the different polarization conditions of the exciting laser. The direction of the polarization is along (c) *x*-axis, (d) *y*-axis, (e) -45°, (f) 45° to the *x*-axis. The brightest fluorescence is observed when the polarization of the excitation light is parallel to the LC director. The cell thickness is 4 μm and the applied field frequency 140 Hz, and the field strength is $V_0 = 40$ V. Scale bar, 10 μm.

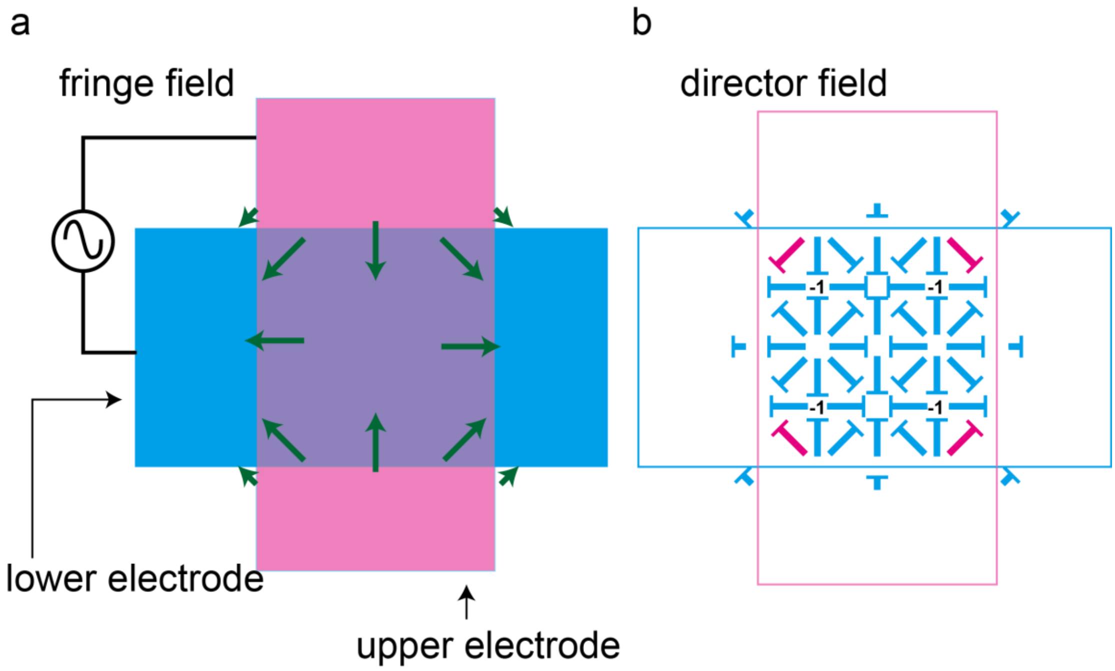

Supplementary Figure 2 : The director field in the square electrode used in this work. (a) Schematic illustration of the fringe electric field. The electric field at the corner of the overlap of the electrodes is always determined uniquely. (b) the corresponding director field. At the corners of the square, the director field is allowed to form -1 umbilics. Thus, even numbers of squares (or odd numbers of umbilics) are generated (for example, 2×2 squares (3×3 umbilics) as shown in (b)).

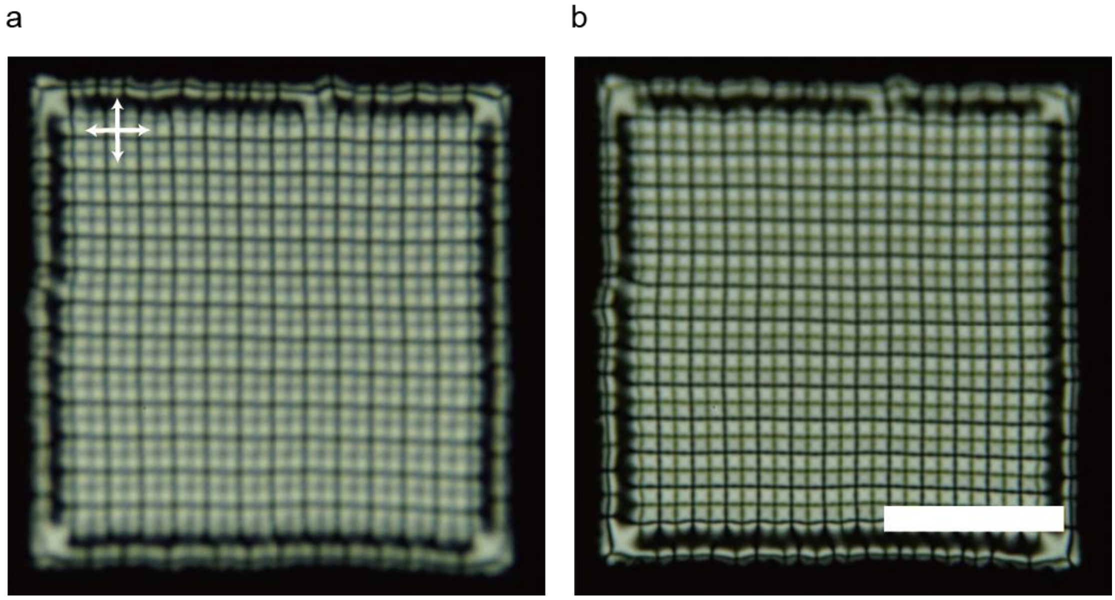

Supplementary Figure 3 : Lens effect of the grid-like texture. The sample cell is positioned (a) close to or (b) far apart from the focus of the objective lens. It can be seen that the contrast of the lines of grids are opposite each other. It is to be noted that the contrast can be inverted if the cell is flipped and the lens effect also changes depending on the height of the condenser lens. The cell thickness, $d = 4.9$ μm. Scale bar, 200 μm.

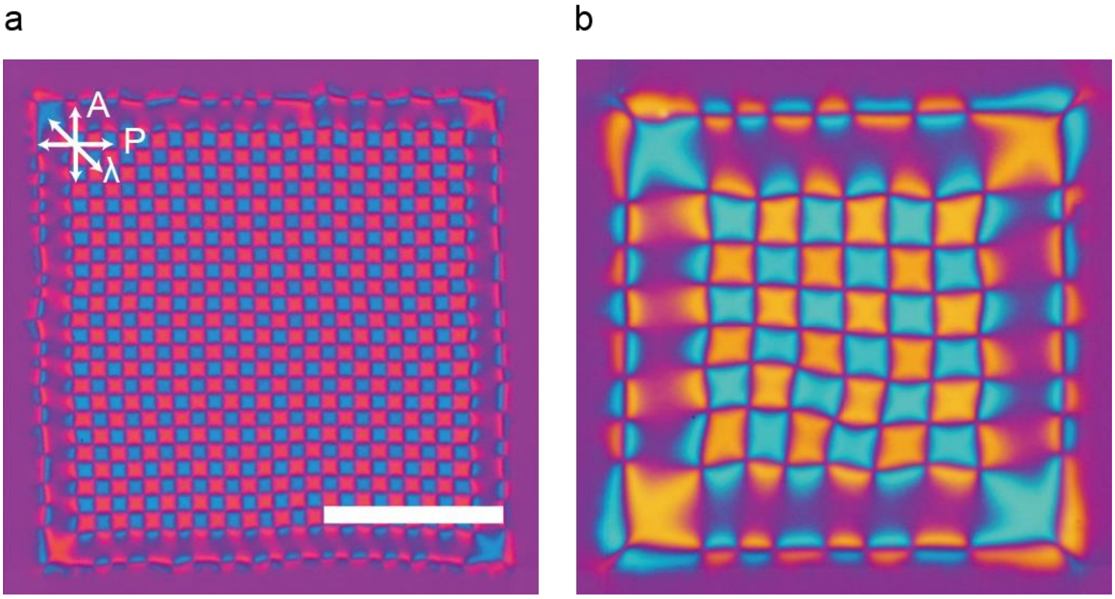

Supplementary Figure 4 : Grid-like textures observed in different cell thickness. (a) and (b) are obtained for a cell thickness of 4.9 μm and 11.4 μm, respectively. The NLC used is CCN-37. The applied voltage is $V_0 = 39.4\,\mathrm{V}$ for both cases. The frequency is adjusted separately. Scale bar, 200 μm.

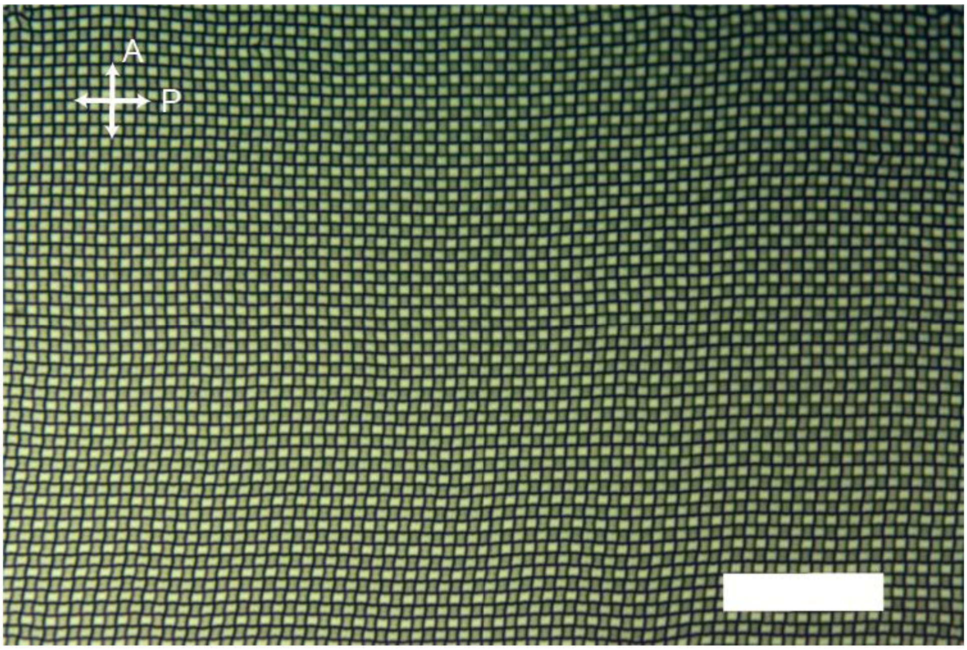

Supplementary Figure 5 : Highly packed defects in a thin cell. A large single domain is prepared by the help of an optical manipulation for a cell thickness of 3.0 μm. The NLC sample is the 1:1 mixture of CCN-47 and CCN-55. The applied voltage is $V_0 = 17.5$ V and the frequency is adjusted to maintain the G state. Scale bar, 100 μm.

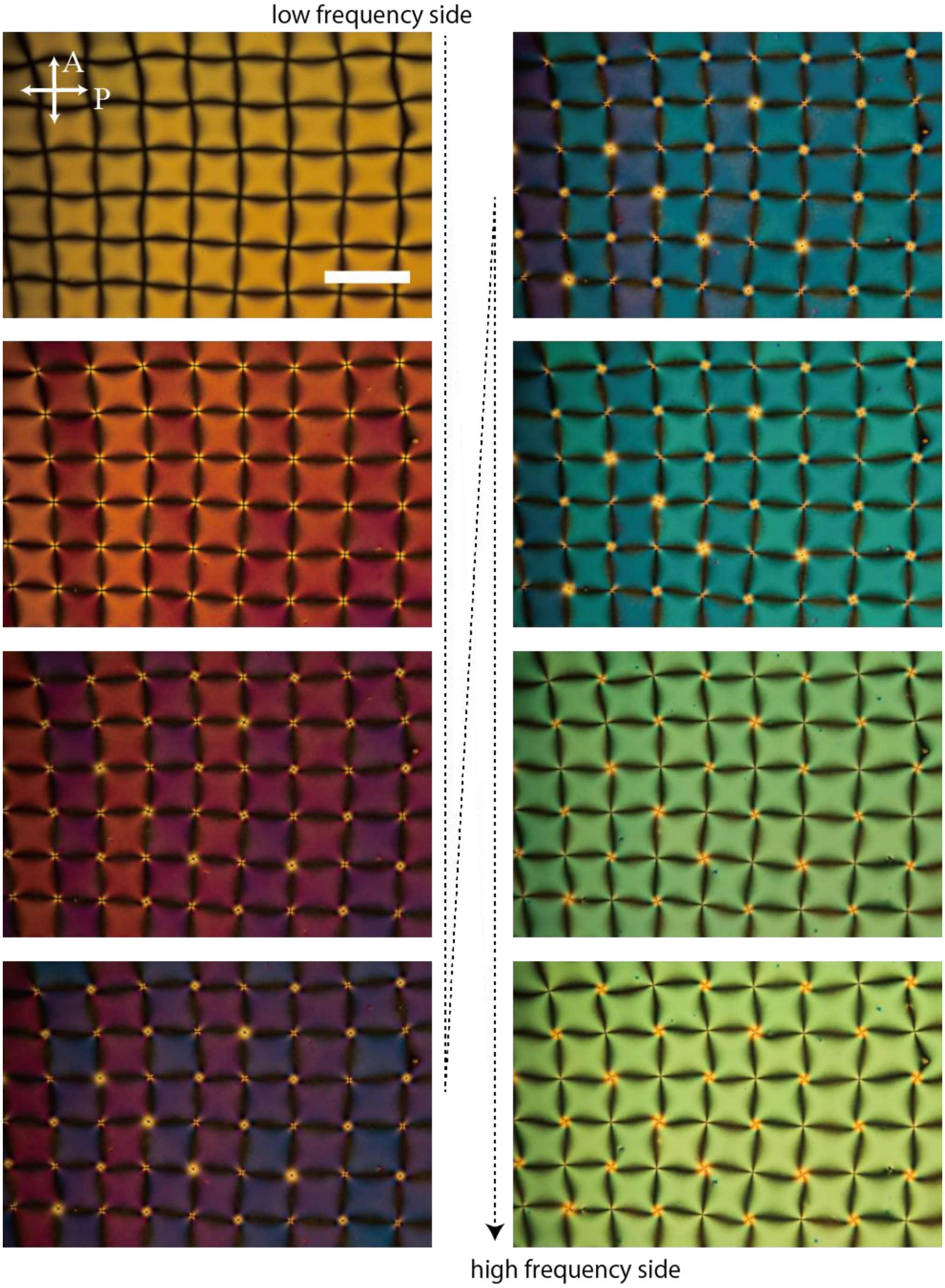

Supplementary Figure 6: Color alternation of the grid-like texture for a thick cell. The frequency is increased in order to tilt the director field to the horizontal plane. The cell thickness is 24 μm. The applied voltage is $V_0 = 17.5$ V. Scale bar, 100 μm. The NLC sample is the 1:1 mixture of CCN-47 and CCN-55.
